# Emergency Contraception: Knowledge and Attitudes of Family Physicians of a Teaching Hospital, Karachi, Pakistan

**DOI:** 10.3329/jhpn.v27i3.3376

**Published:** 2009-06

**Authors:** Hamza M. Abdulghani, Syed I. Karim, Farhana Irfan

**Affiliations:** ^1^ Department of Family and Community Medicine, College of Medicine, King Saud University, PO Box 230155, Riyadh 11321, Saudi Arabia; ^2^ Department of Family Medicine, Aga Khan University Hospital, Karachi, Pakistan

**Keywords:** Cross-sectional studies, Descriptive studies, Emergency contraception, Family physician, Family planning, Knowledge, attitudes, practice, Perceptions, Pakistan

## Abstract

This study was conducted to assess the knowledge of family medicine providers and their attitudes towards emergency contraception in a teaching hospital in Karachi, Pakistan. A 21-item questionnaire containing the demographic profile of respondents and questions concerning knowledge of and attitudes towards emergency contraception was distributed among participants. In total, 45 interviews were conducted, with a response rate of 100%, with faculty physicians (33%), residents (27%), medical officers (40%), 36% male and 64% female physicians; of them, the majority (64%) were married. Although the large majority (71%) of the respondents reported considerable familiarity with emergency contraception, objective assessment revealed deficiencies in their knowledge. About 38% of the participants incorrectly chose menstrual irregularity as the most common side-effect of progestin-only emergency contraception pills, and only 33% answered that emergency contraception was not an abortifacient while 42% were unsure. Forty percent of the physicians prescribed emergency contraception in the past. The large majority (71%) of the physicians were familiar with emergency contraception, yet deficiencies in knowledge inaccuracies were identified. Barriers to its use were identified as ‘it will promote promiscuity’ (31%), religious/ethical reasons (27%), liability (40%), teratogenicity (44%), and inexperience (40%). Overall attitudes regarding emergency contraception were positive; however, most (82%) physicians were unsatisfied with their current knowledge of emergency contraception, and there was a discrepancy between perceptions of physicians and actual knowledge. Interventions providing education to family physicians regarding emergency contraception is strongly recommended.

## INTRODUCTION

Contraception is one of the major determinants of fertility levels. In the developing world, an estimated 122.7 million women have an unmet need for contraception (1). Almost half of the Asian countries had a contraceptive prevalence of 60% or higher (2). This represents a continuous challenge for governments and agencies concerned about ensuring access to contraceptives. Unplanned/mistimed pregnancies generally result from a high unmet need and ineffective use of contraceptives that end in induced abortions (3). Each year, about 79 million unintended pregnancies occur worldwide (4). According to the new worldwide estimates of abortion rates and trends, the overall abortion rates are almost similar in both developing and developed world. However, unsafe abortions are dominating in developing countries (5).

Abortion is a public-health concern because of its impact on maternal morbidity and mortality. In Pakistan, where only 28% of couples use some form of contraception and the gap between the desire to space/limit births and the contraception usage (33%) is one of the widest in the world, and abortion is often the only choice for couples to deal with an unplanned and unwanted pregnancy (6). An estimated 900 million women who wanted to avoid having a child undergo induced abortion annually in Pakistan. This estimates the annual abortion rate of 29 per 1,000 women aged 15-49 years (7). Although the socioeconomic burden of unintended pregnancies is significant, at the same time it is largely preventable (8). In the cases of unprotected sex or method failure, the knowledge about back-up support and use of emergency contraception is the most important factor to prevent unplanned or mistimed pregnancies.

Post-coital emergency contraception may be defined as the use of a drug or a device to prevent pregnancy after intercourse, which has been shown to be safe and effective (9-12). Sooner the first dose was taken after intercourse, the greater is the effectiveness. No single mechanism of action for emergency contraception has been identified. Some studies reported biochemical changes within endometrium while other studies suggest interference within tubal transport of sperm, egg, or embryo that may result in failure of implantation.

Different methods of emergency contraception, including the use of combination estrogen and progestin, progestin alone, and post-coital insertion of an intrauterine device, are available (13). The popular methods include the administration of two doses of a combination estrogen and progestin pill (Yuzpe method) or two doses of progestin alone taken 12 hours after unprotected intercourse, with estimated efficacies of 57% and 85% respectively (14).

Currently, two 0.75-mg doses of levonorgestrel are licensed in Pakistan for use within 72 hours of unprotected sex. Results of a multicentre trial of the World Health Organization also showed a good efficacy with a single dose of levonorgestrel initiated up to 120 hours after intercourse (15). An intrauterine device (IUD) can be inserted up to five days after the first act of unprotected sex. Progestin-only pills reduce the chance of pregnancy by 85%, and combined hormone emergency contraception pills by 57% when taken within 72 hours of unprotected sex. Insertion of copper-T (IUD) reduces the chance of pregnancy up to 99% (16). Despite being effective and safe, emergency contraception is still not widely used (17). The first step towards understanding its use is assessing local physicians' knowledge of the methods and willingness to prescribe them. Previous studies have identified lack of awareness as the most notable barrier to the use of emergency contraception (18,19). Research has examined knowledge, attitudes, and practice patterns of obstetrician-gynaecologists (20), paediatricians, and family-planning specialists with respect to emergency contraception (21).

An extensive literature search in the Internet has failed to show any study from Pakistan on emergency contraception to indicate the attitudes of family physicians. To the best of our knowledge, no study has specifically examined family medicine faculty physicians, residents, and medical officers as an individual group in an academic setting where educational and training issues are particularly pertinent.

The objectives of this study were to survey faculty, residents, and medical officers at a family medicine residency programme with regard to their current knowledge and attitudes towards emergency contraception and to identify barriers to its use.

## MATERIALS AND METHODS

### Study design and subjects

This cross-sectional descriptive study was conducted in the family medicine clinics at the Aga Khan University Hospital (AKUH) and out-campus centers. All faculty physicians, residents, and medical officers in the Department of Family Medicine, AKUH, took part in the study. These participants provide care at nine affiliated clinics in the city and the Family Practice Centre at AKUH. The inclusion criteria were set to include those family physicians who were practising at the Family Practice Centre at AKUH and family practice out-campus (centres).

### Data-collection procedure

Data were collected on a structured pre-coded 21-item questionnaire. We used the Epi Info software (version 6.04) for data entry and the SPSS software (version 12.0) for analysis of data.

### Survey instrument

The survey instrument consisted of a two-page questionnaire developed specifically for this study by the authors through extensive search in similar studies elsewhere. This questionnaire had questions on various aspects of emergency contraception, including knowledge, attitudes, and behaviours. In the first part of the questionnaire, we collected demographic information of the participants. The second part consisted of questions about knowledge, attitudes, and practices relating to emergency contraception and barriers to its use.

### Procedure

Each participant was given the 21-question survey questionnaire and provided an option not to participate. Additional ethical requirements concerning informed consent and confidentiality were ensured by including a paragraph of informed consent at the beginning of the questionnaire.

## RESULTS

### Demographic data of participants

In total, 45 interviews were conducted, with the response rate of 100%, which included faculty physicians (33%), residents (27%), and medical officers (40%). Sixty-four percent were female respondents. The majority (64%) of the respondents were married and had children (72%). This distribution of the respondents was fairly representative of the department (Table 1).

**Table 1.** Demographic profile of study population (n=45)

Demographic profile	No.	Percentage
Position		
Faculty member	15	33
Resident	12	27
Medical officer	18	40
Gender		
Female	29	64
Male	16	36
Marital status		
Married	29	64
Unmarried	16	36
With children	21	72
Without children	6	28

### Knowledge, attitudes, and beliefs

About one-third (29%) of family physicians either did not know anything about emergency contraception or were unsure about the methods while 71% had considerable familiarity with emergency contraception. Only 40% of the respondents correctly chose menstrual irregularity not being most common side-effect of progestin-only emergency contraception while 60% did not know or were unsure. Only 33% of the respondents answered that emergency contraception is not an abortifacient while 42% were not sure about the mechanism. Other barriers were perceived as being use of emergency contraception that would promote promiscuity (31%), religious or ethical reasons (27%), and liability (44%).

Only 42% felt that emergency contraception is a safe medication, and 58% had significant concerns about side-effects or teratogenicity (Table 2).

**Table 2.** Responses to the items regarding “knowledge, attitudes and beliefs, and perceived barriers” about emergency contraception by respondents (n=45)

Response	Yes (%)	No (%)	Unsure (%)
Knowledge			
Do you know about EC?	71	11	18
The correct time for initiation of POEC is 120 hours (correct answer: 72 hours)	5	73	22
Does research show that EC acts as an abortifacient (correct answer: no)	24	33	42
Is pregnancy test necessary before prescribing EC? (correct answer: no)	22	67	11
Is menstrual irregularity is the most common side-effect of EC? (correct answer: no)	38	40	23
After fertilization, can an IUD be effective for EC? (correct answer: yes)	38	47	15
Should POEC be repeated if a woman vomits within two hours? (correct answer: yes)	49	33	18
Did you have an opportunity to learn about EC?	51	49	2
Attitudes and beliefs			
Have you had an opportunity to prescribe EC?	40	60	
Do you feel that benefits of EC outweigh the risks?	58	22	20
Is EC appropriate for discussion at routine consultation?	38	53	9
Does EC-use discourage regular contraceptive-use?	40	53	7
Are you satisfied with your current knowledge of EC?	16	82	2
Would you refer a case to gynaecologist for prescription of EC?	16	80	4
Are you interested in learning more about EC?	96	4	
Should EC be more widely advertised?	44	47	9
Perceived barriers			
Does EC-use promote promiscuity?	31	42	27
Do you feel uncomfortable prescribing EC for religious/ethical reasons?	27	69	4
Are you concerned about liability when you prescribe EC?	40	44	17
Are you concerned about birth-defects/side-effects?	44	42	14
Are you reluctant to prescribe EC because of inexperience with its use?	40	60	


EC=Emergency contraception; IUD=Intrauterine device; POEC: Progestin-only emergency contraception

## DISCUSSION

This study confirmed the findings of several previous studies which have shown clear gaps in knowledge regarding emergency contraception among healthcare providers, including physicians (22-24), nurses (25), paediatricians (26,27), family-planning service providers, and family physicians (17,21). This may affect provision of emergency contraception since they are involved in management, and incomplete knowledge could delay timely scheduling or administration.

The results of the present study demonstrated that the large majority (71%) of family physicians in our institution do have some knowledge; however, only 40% have actually prescribed emergency contraception. Twenty-nine percent of the respondents did not either know or were unsure about the concept of emergency contraception compared to general practitioners in North India where only 41% were vaguely familiar with the concept (18), and 26% of healthcare providers in Turkey who did not know anything about emergency contraception, the method was scarcely known or used (18,28). Objective assessment revealed deficiencies in their knowledge similar to those found in prior studies.

Emergency contraception has been found to be safe and effective (16,29). There is a common misconception that emergency contraception is an abortifacient. Only 33% of the study subjects answered that emergency contraception is not an abortifacient while 42% were unsure. This was similar to the findings of a study by Uzuner *et al.* (17). Previous research indicates that the primary mode of action of emergency contraception is via preimplantation mechanism. Emergency contraception, thus, needs to be positioned as an option distinct from abortion. Emergency contraception is a way to prevent the need for abortion for those who knew about it. Forty-seven percent of the study participants thought that insertion of an IUD after fertilization cannot be effective to prevent pregnancy.

The majority (60%) of the medical practitioners had (favourable) attitudes and supported the availability and use of emergency contraception. More than half (63%) of the participants felt that emergency contraception was not an appropriate topic to discuss at routine consultation. The majority (69%) of the physicians were uncomfortable because of religious reason which is similar to the findings of previous research (19,30) and, therefore, seldom inform or prescribe emergency contraception. Forty-eight percent of the respondents did not had an opportunity to learn about it. About 31% of the (practising) physicians were reluctant to prescribe it because of inexperience with its use and felt uncomfortable (counselling) patients about emergency contraception pills. This finding has significant implications. First, those women who need to use these methods may not be able to obtain adequate information from family physicians. Second, they may not provide adequate information during counselling. The opportunity to initiate emergency contraception is time-limited, and therefore, using it soon after unprotected intercourse is critical to its effectiveness. Women must know about it before they need it or quickly upon identification of need. Lower levels of prescription have been found in studies in developing countries. In Nairobi, Kenya, 15% of family-planning service providers reported having prescribed emergency contraception (28), and 20% of primary healthcare workers recommended emergency contraception in Turkey (31,32). Physicians who are uncomfortable prescribing emergency contraception can still refer cases to another service provider.

Regarding the satisfaction level with their current knowledge, 82% of the physicians were not satisfied, and 96% were interested in learning more about emergency contraception. Veloudis and co-workers reported that physicians must be knowledgeable and be able to educate their patients on contraceptive alternatives (33). Since cases rely on physicians for information on birth control, physicians can improve the knowledge of their service-seekers about emergency contraceptive pills.

This study showed clear gaps in knowledge among family physicians. One way to improve their knowledge is to review and strengthen the curricula of training programmes and continuing medical education activities. Ideally, education of physicians about emergency contraception should occur during their training and should be a regular part of their curriculum, also suggested by others (33).

In the interim, short courses should be provided. We as physicians should encourage and learn to identify our actual learning needs. This issue deserves further exploration and special attention when educational strategies are being designed.

Although it is a small study with a small sample size, further studies, at both state and regional levels, can identify geographic and demographic gaps in family-planning practices. It would also be advisable to examine the knowledge and practices of other healthcare providers, such as nurses, who may offer emergency contraception. These findings show the importance and difficulty in keeping abreast of technological advances, particularly in reproductive technologies which change so rapidly.

### Limitations

There are several limitations in this study. First, the results were generated in one residency programme and may not be generilizable to other programmes or to non-academic settings. This was the first study about knowledge of family physicians and their attitudes towards emergency contraception. No similar study conducted in Pakistan was found for comparison. There may have been some acceptability bias among provider-respondents. Research in the community setting in particular would provide a broader understanding of family physicians with a more exclusive focus on clinical care. However, attitudes and practices relating to emergency contraception may differ among physicians.

The key finding of this study is that academic clinicians have deficiencies in knowledge concerning emergency contraception. The majority of the participants reported that they were unsatisfied with the current knowledge of emergency contraception and interested in learning more about it. Proper training is needed to ensure that physicians are comfortable enough with different methods of emergency contraception to prescribe it when the situation warrants. Educational efforts should be focused on training of healthcare providers to improve correct access of women and effective use of different emergency contraception methods. Discussion about emergency contraception should be raised during routine health check-up visits of women. Future research should be directed at implementing interventions to enhance these types of discussions.

## ACKNOWLEDGEMENTS

The authors thank all family physicians for their participation in the study. They also thank Dr. Sheikh Shafi Ahmad, Assistant Professor and Consultant Statistician in the Department of Family and Community Medicine, for his valuable advice on statistical analysis.
